# Importation of cats and risk of parasite spread: a Caribbean perspective and case study from St Kitts

**DOI:** 10.1186/s13071-020-04365-y

**Published:** 2020-09-23

**Authors:** Jennifer Ketzis, Helle Bork-Larsen, Jernea Bustria, Anne Conan

**Affiliations:** 1 Center for Integrative Mammalian Research, Ross University School of Veterinary Medicine, Basseterre, St Kitts and Nevis; 2 Center for Conservation Medicine and Ecosystem Health, Ross University School of Veterinary Medicine, Basseterre, St Kitts and Nevis

**Keywords:** Cat, Nematode, Trematode, Mite, Spatial movement, Prevalence, Tropical and subtropical regions

## Abstract

**Background:**

In more recent years, international travel with cats has increased. The distribution of cat parasites can change with this movement. Already, subtropical and tropical parasites have been reported by veterinarians in areas where they are not naturally present. Understanding the prevalence of tropical and subtropical parasites in Caribbean islands and the risk of importation to temperate areas could enable improved prevention recommendations and border control import requirements.

**Methods:**

We present a study focused on cat owning students enrolled in a Doctor of Veterinary Medicine (DVM) programme on St Kitts. Owners were interviewed about their cats and their use of parasiticides. Cats were examined for *Trichuris felis* and *Platynosomum fastosum* using sugar flotation, *Lynxacarus radovskyi* using an adhesive tape test, and *Dirofilaria immitis* using commercial antigen and antibody tests.

**Results:**

Data on 115 cats owned by 87 DVM students were collected and 90 cats, all expected to travel to the USA, were examined. Most of the cats were adults and born in St Kitts. Prevalence was reported as 6.8% (95% confidence interval (CI): 2.2–15.1%) for *T. felis*, 16.2% (95% CI: 8.7–26.6%) for *P. fastosum* and 6.8% (95% CI: 2.5–14.3%) for *L. radovskyi*. All *D. immitis* tests were negative. DVM students reported a high level of deworming (83.3% of the cats), but the number of cats treated per recommendations were low (56.1% for endoparasites and 70.8% for ectoparasites). Also, there was a lack of clarity regarding the purpose of the treatments used and treatments did not appear to be targeted for the parasites present.

**Conclusion:**

Our results indicate a low prevalence of the parasites of interest in the DVM student cat population compared to other prevalence studies from the Caribbean. However, a degree of non-compliance with parasiticide uses and the high number of cats traveling to the USA indicate a medium risk of importation of tropical and subtropical cat parasites to temperate areas. We recommend stronger health inspections and health screening requirements at the borders including the development of specific parasiticide protocols for cat importation. 
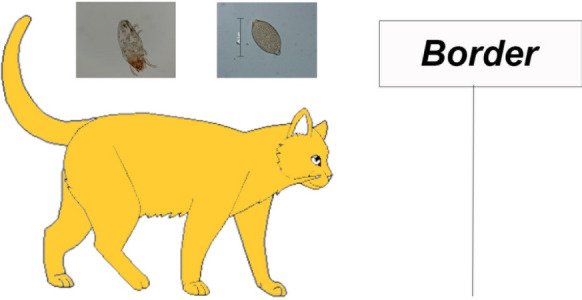

## Introduction

In the last decades, movement of people for tourism and emigration has increased. With emigration, movement of pets between countries and continents is frequent. Concurrently, travel with pets during holidays has increased. While previously travel was with dogs, the trend now is also to travel with cats. Not only do dogs and cats travel with their owners, there are increasing numbers of cats and dogs being imported to be rehomed [1–3]. Case reports of imported ill pets have been published from various countries and veterinarians are diagnosing tropical infectious diseases, in geographical locations where they do not typically occur [4]. In most cases, these are only single occurrences of non-endemic diseases in cats and dogs that have travelled or been recently imported. However, there is the risk of pathogens, such as parasites, becoming established in new regions and the change in distribution of some parasites has been attributed to pet movement [5, 6].

While one might assume that tropical and subtropical pathogens would not become established in temperate climates and *vice versa*, this assumption lacks validity for many parasites. First, the climatic restrictions are not well understood for some parasites and parasites can adapt to different climates. Secondly, temperate climate countries and continents can have subtropical type regions (e.g., Florida in North America and the Mediterranean in Europe). Thirdly, while the impact of global warming on parasite distribution is still being assessed, it is generally accepted that there will be changes in distribution [5, 7]. These changes in distribution have already been seen with some parasites.

A first step in better understanding the potential impact of the increasing travel and importation of cats and dogs on changes in parasite distribution is to obtain data on both the prevalence of parasites in different regions and to assess the parasites in pets that are travelling. This could assist in designing export endo- and ectoparasiticide treatment protocols.

This paper focuses on cats in the Caribbean. While there is, in general, limited prevalence data for the region, there have been recent studies from St Kitts, a Leeward Island, Grenada, a Windward Island and reviews and studies of flea and tick-borne diseases [8–13]. There are also historical studies and case reports from several of the Caribbean islands, providing an overview of the parasites present within the region. Helminths in cats in the Caribbean found in these studies are presented in Table 1 and suggest that while prevalence might differ, the Caribbean islands are relatively consistent in the presence of subtropical and tropical parasites in cats, particularly *Trichuris* sp. and *Platynosomum* sp. In addition, *Dirofiliaria immitis* is endemic on the islands, though few prevalence studies for cats are available [14–16].

**Table 1:** Review of information about presence and prevalence of helminths found in cats in the Caribbean Islands and Countries

Island/Country	Year of study	Study population	Tropical/subtropical parasites (% positive)	Other parasites (% positive)	References
Aruba	1974	18 cats	*Trichuris* sp. (17%)	*Ancylostoma tubaeforme* (28%)	Rep (1975) [34]
				*Oncicola canis* (11%)	
				*Dipylidium caninum* (33%)	
				*Taenia taeniaformis* (17%)	
Bahamas	1957–1958	21 cats	*Trichuris* sp.	*Ancylostoma* spp.	Clarkson & Owen (1959) [35]
				*Eucoleus* (*Capillaria*) *aerophila*	
				*Physaloptera praeputialis*	
				*Toxocara cati*	
				*Dipylidium caninum*	
				*Taenia taeniaformis*	
Bonaire	1974	12 cats	*Trichuris* sp. (25%)	*A. tubaeforme* (42%)	Rep (1975) [34]
				*O. canis* (42%)	
				*Thelazia* sp. (17%)	
				*D. caninum* (58%)	
				*T. taeniaformis* (25%)	
Grand Cayman	2009–2010	55 free-roaming cats^a^	*Platynosomum fastosum* (29%: 2% active infection; 27% resolving infections)	*Spirometra* spp. (18%)	Headley et al. (2012) [36]
	2013–2014	36 free-roaming cats^a^	Not tested	*Dirofilaria immitis* (11%)	Darby et al. (2019) [15]
Curaçao	1974	53 cats	*Trichuris* sp. (23%)	*A. tubaeforme* (83%)	Rep (1975) [34]
			*P. fastosum* (4%)	*O. canis* (2%)	
				*T. cati* (4%)	
				*Thelazia* sp. (4%)	
				*D. caninum* (26%)	
				*Spirometra mansonoides* (4%)	
				*T. taeniaformis* (42%)	
Grenada	2004–2008	137 free-roaming cats^a^	Not tested	*D. immitis* (9%)	Fernandez et al. (2010) [37]
	2018	free-roaming^a^ and domestic cats	*Trichuris* sp. (33%)	*Ancylostoma* sp. (79%)	Paterson et al. (2019) [16]
			*Mammomonogamus* sp. (40%)	*D. immitis* (14%)	
		57 fecal examinations		*Toxocara cati* (9%)	
				*Toxascaris leonina* (2%)	
		65 blood samples		*D. caninum* (3.5%)	
Guadeloupe	1982–1983	31 cats	Not detected	*Ancylostoma* sp.	Esterre & Maitre (1985) [38]
				*T. cati*	
				*T. leonina*	
				*T. taeniaeformis*	
Jamaica	1953^c^	Case report	*P. fastosum*	*P. praeputialis*	Guilbride (1953) [39]
			*Mammomonogamus* (*Syngamus*) *ierei*		
Puerto Rico	1964	70 cats from San Juan	*Trichuris vulpis* (6%)	*A. tubaeforme* (51%)	de León & Kolodziej (1969) [40]
			*P. fastosum* (72%)	*A. braziliense* (85%)	
				*O. canis* (2%)	
				*P. praeputialis* (3%)	
				*T. cati* (75%)	
				*D. caninum* (42%)	
				*Diphyllobothrium mansoni* (10%)	
				*T. taeniaeformis* (3%)	
	1973	5 cats from Ponce	*P. fastosum*	*A. tubaeforme*	Acholonu (1977) [41]
				*P. praeputialis*	
				*D. caninum*	
				*Spirometra mansoni*	
				*T. taeniaeformis*	
St Kitts	2005–2006	100 free-roaming cats^a^	*Trichuris* sp. (71%)	Hookworms (88%)	Krecek et al. (2010) [12]
			*Platynosomum* sp. (81%)	*Eucoleus aerophilus*	
			*Mammomonogamus* spp. (45%)	*Physaloptera* sp.	
				*Toxocara* spp.	
				Taeniid	
	2013–2014	41 owned indoor and indoor/outdoor cats	*Trichuris* sp. (26%)	*Ancylostoma* sp. (5%)	Ketzis et al. (2015) [8]
			*Platynosomum* sp. (22%)	*T. cati* (2%)	
			*Mammomonogamus* spp. (2%)		
	2014–2015	35 free-roaming cats^a^	*Trichuris felis* (83%)	*A. tubaeforme* (94%)	Geng et al. (2018) [10]
			*P. fastosum* (57%)	Capillarids (17%)	
			*M. ierei* (57%)	*T. cati* (9%)	
				*D. caninum* and *T. taeniaformis*) (31%)	
	2017	50 free-roaming cats^a^	*Trichuris* sp. (66%)	*A. tubaeforme* (88%)	Eisenbraun et al. (2019) [9]
			*P. fastosum* (68%)	Capillarids (2%)	
			*M. ierei* (60%)	Metastrongyloid larvae (2%)	
				*Physaloptera* sp. (2%)	
				*T. cati* (16%)	
				*D. caninum* (2%)	
				Taeniids (14%)	
Trinidad and Tobago	2013	Case report	*P. fastosum*	–	Montserin et al. (2013) [42]

^a^Free-roaming refers to unowned cats

^b^Year of publication; date of study not indicated

St Kitts, with the most data on helminths in cats, also hosts the USA accredited school, Ross University School of Veterinary Medicine (RUSVM). The RUSVM Doctor of Veterinary Medicine (DVM) programme consists of 7 ‘semesters’ (28 months total) of a preclinical programme during which the students are resident on St Kitts. This is followed by 12 months of a clinical programme based primarily at universities in the USA, although students also can opt to complete this programme at affiliated schools in Canada, the UK, Ireland and Australia. Many DVM students bring their pets with them when they attend their clinical year. In addition, during their studies, some travel back and forth to the USA with their pets. This DVM student-owned cat population provides an opportunity to determine the prevalence of parasites in cats destined to travel to the USA or elsewhere, as well as student practices in term of parasite control. In this study, examinations of these cats focused on three parasites known to have high prevalence on St Kitts [8, 9, 17]: *Trichuris felis* (proposed syn. *T. serrata*, *T. campanula*), *Platynosomum fastosum* (syn. *P. concinnum*) and *Lynxacarus radovskyi. Dirofilaria immitis* also was included since microfilaria-positive cats are not rare on the island. While these parasites are present in some areas of the USA, importation of infected cats to other areas could be a risk for establishment in a new location.

## Methods

In this study, the target population was the cats owned by RUSVM DVM students during their preclinical training on St Kitts. Before interviews and sampling, information about the project was provided through campus communications. Any DVM students owning a cat could participate in the study. Sampling was convenient, with students volunteering to participate. Students who agreed to answer the questionnaire were not compelled to bring their cat(s) for sampling. Therefore, we expected to interview 150 DVM students and sample 100 cats.

After obtaining informed consent, the students were interviewed using a standardized questionnaire to obtain information about cat demography, spatial origin and medical history. Information about recent and future travel to the USA was also collected. DVM students in 6th and 7th semester were contacted in the 2 months before their departure from St Kitts to attend their clinical year and were asked to complete a second questionnaire about the future life of their cats. Participants were then asked to bring their cat to the Ross University Veterinary Clinic (RUVC) to obtain the requested samples.

Feces, blood and hair were collected to assess the endo- and ectoparasites of each cat. Fresh feces were collected by the owner from the litter box and brought to the investigator the day of the scheduled visit. If the cat could go outdoor, the owner was requested to keep the cat inside until fresh feces could be obtained. Feces were to be collected the day of the scheduled visit. If this was not feasible, feces were to be collected as soon as feasible after the visit and brought within 24 h of collection to the investigator. During the scheduled visit, after a basic health examination, blood and hair samples were collected. A maximum of 2 ml of blood was drawn from the jugular or saphenous vein. If insufficient or no blood was obtained after three attempts, the blood sampling was abandoned as per the Institutional Animal Care and Use Committee (IACUC) approved protocol. Five hair samples were collected using adhesive tape following the technique described in Ketzis et al. [17]. Cats were not excluded from the study in the absence of one or two of the three samples (feces, blood or hair).

Fecal samples were stored for up to 5 days at 4–8 °C prior to analysis by double centrifugation. In brief, 1 g of feces was mixed with approximately 20 ml of water and centrifuged for 5 min at 500×*g*. The pellet obtained was resuspended in Sheather’s sugar flotation solution (specific gravity 1.27–1.28), centrifuged with a coverslip for 5 min at 500× *g* and allowed to sit for an additional 10 min prior to examining the coverslip at 100× magnification. All parasitic organisms on the coverslip were counted and recorded. Blood was analyzed using the IDEXX Snap Feline Triple Test® and the Heska Solo Step Feline Heartworm Test® on the day of the examination. Hair samples were examined on the day of collection at 40–120× magnification for the presence of *L. radovski.*

Data from the sampling were entered in an excel spreadsheet. Both questionnaires were filled using Epi Info^TM^ [18]. All data were then imported in R software for data management and data analyses. Data analyses consisted of description only. Confidence intervals at 95% (95% CI) were calculated using the function ‘ci.binomial’ in the package *epiDisplay* [19].

## Results

From February to October 2018, 87 students were interviewed about their cats. Data on 115 cats were obtained. Numbers of male and female cats were equivalent and the majority of cats were adults (*n* = 64). Most of the cats (*n* = 92) were born in St Kitts and adopted by students on the island. Few cats (*n* = 13) were reported as having chronic diseases (Feline Immunodeficiency Virus, feline asthma, hypothyroidism, etc.) and 12 had already been diagnosed with an infectious disease. Only two students reported a previous diagnosis of *T. felis* (*n* = 1) or *P. fastosum* (*n* = 2). Cat demographic information is provided in Table 2.

**Table 2:** Demographic description (sex, age and origin) of the studied cat population

	DVM student-owned cats, *n* (%)	Sampled cats, *n* (%)
Male		
Kitten (< 6 months)	8 (7.0)	4 (4.4)
Young (6–11 months)	12 (10.4)	10 (11.1)
Adult (1–6 years)	35 (30.4)	28 (31.1)
Senior (7+ years)	3 (2.6)	3 (3.3)
Female		
Kitten (< 6 months)	12 (10.4)	8 (8.9)
Young (6–11 months)	12 (10.4)	6 (6.7)
Adult (1–6 years)	29 (25.2)	28 (31.1)
Senior (7+ years)	4 (3.5)	3 (3.3)
Place of birth (Missing value: 1)		
St Kitts	92 (88.5)	69 (77.5)
USA	20 (19.2)	18 (17.3)
Canada	2 (1.9)	2 (1.9)

*Notes*: “DVM student-owned cats” column describes the cat population of DVM students who answered the questionnaire while the “sampled cats” column describes the cats from which samples were collected

A total of 90 of these cats (78.3%) were sampled between February and October 2018. Fecal samples, hair samples and blood samples were obtained for 74, 88 and 74 cats respectively. *Platynosomum fastosum* was identified in the feces of the most cats with a prevalence of 16.2% (95% CI: 8.7–26.6%; *n* = 12). Prevalence of *T. felis* was 6.8% (95% CI: 2.2–15.1%; *n* = 6) and for *L. radovskyi* was 6.8% (95% CI: 2.5–14.3%; *n* = 6) (Table 3). Prevalences for *P. fastosum*, *T. felis* and *L. radovskyi* in male cats were 20% (95% CI: 7.7–38.6%; n=6/30), 8.3% (95% CI: 1.8–22.5%; *n* = 3/36) and 2.2% (95% CI: 0.1–11.8%; *n* = 1/45), respectively; prevalences in female cats were 15.8% (95% CI: 6.0–31.2%; *n* = 6/38), 5.3% (95% CI: 0.6–17.8%; *n* = 2/38) and 11.6% (95% CI: 3.9–25.1%; *n* = 5/43). Kittens, young cats and adult cats were infected with *P. fastosum* (*n* = 1, *n* = 2 and *n* = 9 respectively), *T. felis* (*n* = 2, *n* = 1 and *n* = 2) and *L. radovskyi* (*n* = 1, *n* = 1 and *n* = 4). All samples from senior cats were negative for *P. fastosum* (*n* = 0/6), *T. felis* (*n* = 0/6) and *L. radovskyi* (*n* = 0/5). Two cats had co-infection with the parasites of interest: *T. felis* + *P. fastosum* (*n* = 1), *T. felis* + *L. radovskyi* (*n* = 1). All animals tested for *D. immitis* (*n* = 74) were antibody- and antigen-negative (Table 3). The use of endo- and ectoparasiticide treatment is summarized in Table 4.

**Table 3:** Prevalence of parasites in DVM student-owned cats

Species	Positive samples	Prevalence (%)	95% CI
Parasites of interest			
*Trichuris felis*	5	6.8	2.2–15.1
*Platynosomum fastosum*	12	16.2	8.7–26.6
*Lynxacarus radovskyi*	6	6.8	2.5–14.3
*Dirofilaria immitis*	0	0	0–4.9
Other parasites			
*Ancylostoma* sp.	11	14.8	7.7–25.0
*Toxocara cati*	2	6.8	2.2–15.1
Coccidia	9	12.2	5.7–21.8
Taeniid eggs	2	6.8	2.2–15.1
*Felicola subrostratus*	1 (eggs not confirmed)	–	–
*Cheyletiella* spp.	2	2.3 %	0.3–8.0

*Notes*: Total sampled cats: 90 (74 fecal flotation, 88 hair tape and 74 blood samples)

*Abbreviation*: 95% CI, 95% exact binomial confidence intervals

**Table 4:** Use of ecto- and endo-parasiticide treatments in DVM student-owned cats

	DVM student-owned cats (*N* = 114)	Cats positive for *Trichuris* sp. and/or *Platynosomum fastosum* (*N* = 16)	Cats positive for *Lynxacarus radovskyi* (*N* = 6)
	*n* (%)	*n* (%)	*n* (%)
Dewormed at least once during the last year	95 (83.3)	11 (68.8)	–
Plan to use an endoparasite product during the next year	50^a^ (45)	8 (50)	–
Plan to use an ectoparasite product during the next year	102 (89.5)	–	5 (83.3)
Monthly heartworm preventative	61^b^ (56.0)	7^f^ (46.7)	–
Treated for ectoparasites at least once	96 (84.2)	–	4 (66.7)
Product listed is correct for the use (open-ended question)	42^c^ (42.0)	8^g^ (61.5)	4^h^ (80)
Treated per recommendations for endoparasites	60^d^ (56.1)	7^f^ (46.7)	–
Treated per recommendations for ectoparasites	80^e^ (70.8)	–	4 (66.7)

^a^*N* = 111; ^b^ *N* = 109; ^c^ *N* = 100; ^d^ *N* = 107; ^e^ *N* = 113; ^f^ *N* = 15; ^g^ *N* = 13; ^h^ *N* = 5

Out of 114 cats, six travelled back and forth to the USA with their owner during the owner’s residence on St Kitts. One cat travelled 3 times in less than two years. However, owners of 37 cats were planning to travel back to the USA with their cats in 2018. Reported past and future destinations included 19 USA states or territories.

Eleven students were interviewed before their departure to the USA and all students planned to travel to the USA with their cats. Their destinations included Florida, Illinois, Louisiana, Michigan, Oklahoma, Oregon, Tennessee, Texas and Wisconsin. Two of the cats were positive for *P. fastosum* but treated after diagnosis. There were no cats positive for *T. felis* and *L. radovskyi*. None of the cat owners planned to allow their cats free outdoor access with eight cats to be kept inside at all times and three cats to have access to a balcony or outside only under supervision.

## Discussion

This study confirmed the presence of tropical and subtropical parasites of interest in the RUSVM DVM student-owned cats. The prevalence of *T. felis*, *P. fastosum* and *L. radovskyi* were lower than expected in the student-owned cats. Previous studies in St Kitts showed higher prevalence, 26% for *T. felis* and 22% for *P. fastosum* [8]. Regarding *D. immitis*, given that adult infections are rare in cats compared to dogs, the negative antigen tests were not unexpected [20]. The negative antibody tests might also be due to the spatial heterogeneous distribution of *D. immitis* on St Kitts [21] or the small sample size, especially since over half of the cats were on a heartworm preventative.

While prevalence of the parasites of interest was lower than previously, treatment has been emphasized in the RUSVM DVM programme since the previous study. Unfortunately, the proportion of cats treated following the recommendations (monthly broad-spectrum heartworm preventative and year-round ectoparasite control) is low (56.1% for endoparasites and 70.8% for ectoparasites). Moreover, the prevalence of other parasites such as *Ancylostoma* sp. and *Toxocara cati*, both of which can be zoonotic, indicate that these cats are not dewormed properly. During the investigation, the informal response of owners who did not use parasiticides was often: “my cat does not go outside”. While the risk of infection is indeed lower for cats staying inside, cats can still be infected by some of the parasites such as *P. fastosum.* Not all of the students in the study had completed the parasitology classes at RUSVM, so we were not expecting a full knowledge of preventative use. However, we should consider their knowledge of parasite epidemiology higher than the general population. This underlines that education of any cat owner should be improved to increase the appropriate use of anti-parasiticides and the regular health check of cats by a veterinarian.

Travel to the USA with cats, other than after completion of studies on St Kitts, was not frequent; however, when travel occurred, destinations varied. In some of the travel destinations with suitable climates for subtropical parasites, such as Florida, the parasites studied already occur although at a generally low prevalence [22, 23]. In other destinations with potentially suitable climates, such as Oklahoma, there have been reports of *T. felis*, for example, but only from cats imported from the Caribbean and not in prevalence studies [24, 25]. In addition, cats are being imported to areas where there have been no previous reports of some of the parasites considered in this study.

Our results show a low risk of introduction of parasites to the USA by the DVM students (low prevalence and limited travel back and forth). However, it does show the possibility of the importation of infected cats to areas free of the studied parasites. This study was limited in the focus of the parasites investigated based on the diagnostic methods used. We excluded, for example, *Strongyloides stercoralis*, *Physaloptera* sp., and flea- and tick-borne diseases (e.g., *Babesia*, *Rickettsia*), which are known to occur in the Caribbean and have limited distribution or are absent in the USA [11, 12, 26, 27]. If we consider that DVM students have better practices of parasite prevention than the general population, the prevalence of these parasites found in other studies from the Caribbean, and the limited parasite focus, our results would indicate a medium risk of introduction by the general population traveling from the Caribbean to the USA with their cats.

Most regulations regarding pet travel and importation involve minimal requirements and are focused on notifiable diseases, which pose a potential risk to public health and livestock. For cats, diseases such as rabies, leishmaniosis or anthrax are of interest and reported. The import regulations do not necessarily consider the potential of imported cats introducing new diseases of cat concern only. To enter the USA, cats are only required to pass visual inspection. Neither the USDA APHIS Veterinary Services, the Center for Disease Control and Prevention, nor the U.S. Fish and Wildlife Service have requirements for the importation of domestic cats, although most USA airlines require a health certificate and animals can be inspected at the port of entry. To complicate matters, under emergency evacuation situations, the USA can temporarily suspend import requirements for cats and dogs travelling from impacted areas [28]. In the European Union, rabies vaccination is required for entrance into the Schengen area and, depending on the origin, serology testing is also required. Some countries do require a specific or general endo- and/or ectoparasiticide treatment prior to entry but the treatments are not related to any diagnostics on what the cat/dog might have, efficacy is not evaluated post-treatment and few endo- and ectoparasiticides are registered for the use of treating tropical and subtropical parasites. EU countries free of *Echinococcus multilocularis* (Finland, Republic of Ireland and Malta) require dogs to be treated with praziquantel before entry. Cats do not require treatment despite recent evidence that they play a role in transmission of this cestode [29]. Even in countries with strict requirements such as Iceland where treatment and quarantine are required, a study by Skírnisson et al. [30] indicates that infections can still exist with at least two mite species becoming established despite the strict requirements. Other pathogen movements have been shown, highlighting the lack of awareness of the risk of pathogen transportation. For example, animals rescued after Hurricane Katrina spread *Dirofilaria immitis* to other USA regions [31], recently a case of leishmaniosis occurred in a UK dog who never travelled [32] and changes in the strain and distribution of *Echinococcus multilocularis* in parts of Canada have been attributed primarily to the importation of foxes and dogs from Europe [33].

While products are registered for the prevention of *D. immitis*, no endo- or ectoparasiticide products are registered for the treatment of the other parasites focused on in this study nor for some of the other subtropical and tropical parasites, such as *Mammomonogamus ierei*, known to occur in the region. There are recommended treatments for these parasites in the literature; however, not only are the recommended treatments extra-label, some such as *P. fastosum* which is treated with praziquantel, require higher doses and more frequent administration than the labelled dose for products that contain the effective compound. This suggests that standard deworming protocols are insufficient and that parasite diagnostics prior to export of the cats is needed to tailor the treatment to the parasites present.

## Conclusion

Our results show that specific parasiticide protocols are required and should be emphasized before the importation of cats to the USA. Moreover, a more complete health check should be performed for cats entering the USA.

## Data Availability

Data supporting the conclusions of this article are included within the article. All data generated or analyzed during this study are available from the corresponding author upon request.

## Abbreviations

CI: Confidence interval
DVM: Doctorate in Veterinary Medicine
IACUC: Institutional Animal Care and Use Committee
IRB: Institutional Review Board
RUSVM: Ross University School of Veterinary Medicine
RUVC: Ross University Veterinary Clinic
USDA APHIS: United States Department of Agriculture–Animal and Plant Health Inspection Services
